# Expression analysis of genes encoding *TEX11, TEX12, TEX14* and *TEX15* in testis tissues of men with non-obstructive azoospermia

**DOI:** 10.5935/1518-0557.20180030

**Published:** 2018

**Authors:** Parnaz Borjian Boroujeni, Marjan Sabbaghian, Mehdi Totonchi, Niloofar Sodeifi, Homa Sarkardeh, Azam Samadian, Mohammad Ali Sadighi-Gilani, Hamid Gourabi

**Affiliations:** 1 Department of Genetics, Reproductive Biomedicine Research Center, Royan Institute for Reproductive Biomedicine, ACECR, Tehran, Iran; 2 Department of Andrology, Reproductive Biomedicine Research Center, Royan Institute for Reproductive Biomedicine, ACECR, Tehran, Iran; 3 Department of Stem Cells and Developmental Biology, Cell Science Research Center, Royan Institute for Stem Cell Biology and Technology, ACECR, Tehran, Iran; 4 Department of Urology, Shariati Hospital, Tehran University of Medical Science, Tehran, Iran

**Keywords:** Male infertility, Gene expression, Non-Obstructive Azoospermia, Intercellular bridges, Synaptonemal complex

## Abstract

**Objective:**

Spermatogenesis is a complex process controlled by a plethora of genes. Changes in expression and function of these genes may thus lead to spermatogenic deficiency and male infertility. *TEX11, TEX12, TEX14* and *TEX15* are germ cell-specific genes expressed in the testis. *TEX11*, involved in the initiation and maintenance of chromosome synapses in meiotic chromosomes, has been shown to be essential for meiosis and fertility in males. *TEX14*, a component of intercellular bridges in germ cells, is required for spermatogenesis and fertility. *TEX12* and *TEX15* are essential for correct assembly of the synaptonemal complex and thus meiosis progression.

**Methods:**

In order to examine whether changes in expression of these genes is associated with impaired spermatogenesis, expression levels of these genes were quantified by RT-qPCR on samples retrieved from infertile patients submitted to diagnostic testicular biopsy at Royan institute. Samples were divided into two groups of 18 patients with non-obstructive azoospermia considered as case; nine patients with obstructive azoospermia were included in the control group.

**Results:**

A significant down-regulation of these genes was observed in the SCOS group when compared to the control group.

**Conclusion:**

This result suggests that regular expression of *TEX11, TEX12, TEX14* and *TEX15* is essential for the early stages of spermatogenesis.

## INTRODUCTION

Male-factor infertility apparently accounts for 40% to 50% of infertile complications and may be defined by environmental reasons, infections, immunological or hormonal insufficiencies, while many are regulated by genetic factors (Hirsh, 2003; Brugh & Lipshultz, 2004; Boyle *et al*., 1992; Ma *et al*., 2016). Spermatogenesis is a complex process controlled by thousands of genes, where any change in the expression or function of these genes may lead to spermatogenic failure and male infertility (Zhou *et al*., 2009; Westerveld, 2008). Identification of stage-specific genes controlling spermatogenesis is thus important.

Previous studies have revealed that *TEX11, TEX12, TEX14* and *TEX15* expression is restricted to germ cells and is not detectable in somatic tissues of humans and mice (Wang *et al.*, 2001; Adelman & Petrini, 2008). Multiple studies have shown that X-linked germ cell-specific genes such as testis-expressed gene 11 (*Tex11*) have significant roles in regulating male fertility (Zheng *et al*., 2010; Matzuk & Lamb, 2008). Furthermore, *TEX11* expression has been associated with the onset of spermatogenesis, although restricted to spermatocytes and round spermatids (Wang *et al.*, 2005; Tang *et al*., 2011).

The synaptonemal complex (SC) is a large protein structure needed for synapsis and the successful completion of meiotic cell division. Incorrect assembly of this complex in mice results in maturation arrest and infertility (Bolcun-Filas *et al.*, 2009; Hamer *et al*., 2008; Kouznetsova *et al.*, 2011), and may similarly lead to infertility, recurrent miscarriage and aneuploidies such as Down syndrome in humans (de Vries *et al*., 2005; Gerton & Hawley, 2005; Page & Hawley, 2003). *Tex11*, *Tex12* and *Tex15* are needed in chromosomal synapsis and meiotic recombination. More specifically, *Tex11* forms distinct foci on homologous chromosomes that synapse with each other and seem to be a new component of meiotic nodules needed for recombination. In the absence of this protein, chromosomal synapsis stops and crossover formation is reduced (Stouffs & Lissens, 2012; Yang *et al.*, 2008b). *Tex12* is a meiosis-specific protein essential for the progression of synapsis between homologous chromosomes in male and female germ cells (Hamer *et al*., 2008). Loss of *Tex15* leads to meiotic recombination failure, since this protein is responsible for the transfer of DNA repair proteins onto double strand break (DSB) locations (Yang *et al*., 2008a). *TEX12* is small with no known domains, but has orthologs in other mammals such as mice, cows, and dogs (Hamer *et al*., 2006; Wang *et al*., 2001). *TEX15* has orthologs in mammals and zebra fish (Yang *et al.*, 2008a). It is abundantly expressed in post-meiotic germ cells, spermatogonia, and early spermatocytes, showing that this gene plays a role in different stages of spermatogenesis (Yang *et al*., 2008a; Wang *et al.*, 2005). Interestingly, it is expressed in both the testis and ovaries, as is its mouse ortholog (Wang *et al*., 2001).

Germ cell intercellular bridges are required for fertility in invertebrates (Brill *et al.*, 2000; Robinson *et al.*, 1994; Robinson & Cooley, 1996) and have been preserved from invertebrates to humans (Fawcett *et al*., 1959), indicating their importance in fertility (Greenbaum *et al.*, 2006; 2009). Mammalian intercellular bridges connect hundreds of germ cells in syncytia originated from a single spermatogonial stem cell (Huckins, 1971; Greenbaum *et al.*, 2006; 2009). In the absence of cell bridges in mammalians, syncytium is thus not formed and as a result spermatogenesis is impaired (Greenbaum *et al.*, 2006; 2009). *TEX14* is needed to generate the germ cell intercellular bridges from the midbody (Greenbaum *et al.*, 2006). Human *TEX14* is preferentially expressed in the testis with its highest levels observed in spermatocytes and early round spermatids (Wu *et al*., 2003). Greenbaum *et al.* (2006) showed that *Tex14-/-* mutant male mice do not form intercellular bridges, thus confirming its essentiality in mammalian spermatogenesis. It was later revealed that *Tex14* is also essential for complete spermatogenesis in Finnish Yorkshire boars as in mice (Sironen *et al.*, 2011). Chung *et al*. (2013) recognized new loci showing association with development of testicular germ cell cancer (TGCT), one of which (rs9905704) located in *TEX14*. It has also been shown that common genetic variations in *TEX14*, which is over-expressed in breast tumors, is associated with risk of breast cancer in Caucasians (Kelemen *et al*., 2009). Recent studies showed that although *Tex11-/-*, *Tex14-/-* and *Tex15 -/-* mutant male mice were sterile because of meiotic arrest and disturbance of spermatogenesis, mutant females were fertile. They suggested that the infertility observed in mutant male mice was caused by loss of function of these genes and that *Tex11*, *Tex14* and *Tex15* were needed for meiosis progression and fertility only in male mice (Yang *et al*., 2008a; Greenbaum *et al.*, 2006; Sironen *et al.*, 2011). However, *TEX12* has identical localization patterns in oocytes and spermatocytes, revealing that this protein is a common part of the central element of the synaptonemal complex in mammals and is not sex-specific (Hamer *et al*., 2006). *Tex12 -/-* mutant male mice were azoospermic and therefore sterile because of failure in chromosomal synapsis progression, while *Tex12 -/-* female mice were sterile because of loss of ovarian follicles (Hamer *et al.*, 2008).

The homology of amino acid sequences and expression patterns of the human and murine *TEX11*, *TEX12, TEX14 and TEX15* genes (Wang *et al*., 2001; Zheng *et al*., 2010; Tang *et al*., 2011; Stouffs *et al*., 2009; Greenbaum *et al*., 2006; Wu *et* *al.*, 2003; Hamer *et al*., 2006 ; Yang *et al*., 2008b) suggests that these genes may play some a few in human spermatogenesis. Given that deficiency of these genes results in azoospermic mice (Zheng *et al*., 2010; Greenbaum *et al*., 2006; Wu *et al.,* 2003; Hamer *et al*., 2006; Yang *et al*., 2008a), severe spermatogenic failure may thus be caused by their disruption. Therefore, this study aimed to replicate this finding in humans by examining the expression levels of *TEX11*, *TEX12, TEX14*, and *TEX15* in the testis tissue of patients with non-obstructive azoospermia and comparing them against controls.

## MATERIAL AND METHODS

### Patients

Twenty-seven tissue samples were obtained from 18 patients with non-obstructive azoospermia (NOA) and nine subjects with obstructive azoospermia. All participating infertile men had undergone testicular sperm extraction (TESE) procedures at the Royan Institute. All patients gave written informed consent and the Ethical Review Board of the Royan Institute approved the study (reference number EC/90/ 1050).

Nine of the 18 patients with NOA were diagnosed with Sertoli cell-only syndromes (SCOS), while the other nine were diagnosed with maturation arrest at the spermatocyte stage. The control group consisted of nine patients with obstructive azoospermia. Patients with abnormal karyotypes and Y chromosome microdeletions were excluded from the study.

The age range of the patients at the time of diagnosis was 30 to 50 years and their mean age was 36.91±5.39 years. Hormonal levels were measured using a competitive ELISA kit (Monobind, CA, USA) according to the procedure described by Zangeneh *et al*. (2015). Cytogenetic analysis was performed based on standard methods (Asia *et al.*, 2014).

### Histological evaluation

After TESE, a portion of the testicular samples was submerged in Bouin's solution and sent for standard histopathological analysis (McLachlan *et al*., 2007). Residual tissues were collected by the Royan Tissue Bank. Testicular histopathology was categorized according to the most recognized pattern of spermatogenesis process: samples with complete spermatogenesis (obstructive azoospermia), samples with spermatogenic maturation arrest at the spermatocyte stage or SCOS.

### RNA extraction

Total RNA from frozen testis tissue was extracted with TRIzol (Invitrogen, Carlsbad, CA, USA) according to the manufacturer's instructions. Approximately 50-100 mg of testis tissue was pipetted into one mL of TRIzol. RNA was detached with chloroform, precipitated with isopropanol, washed with 75% ethanol, and finally dissolved in DEPC treated water (Li *et al.*, 2006).

The concentration of RNA samples was determined spectrophotometrically by measuring absorption at 260 nm (Li *et al*., 2006), while DNA and protein contamination were checked by optical density (OD) measurements at 260/280 nm.

The integrity of total RNA was evaluated by measuring 260:280 nm absorption ratios and by gel electrophoresis on 1.2% agarose gel (Yu *et al*., 2007; Tang *et al*., 2006).

### DNase treatment and cDNA synthesis

Before total RNA reverse transcription (RT), the samples were treated with DNase to eliminate DNA contamination using the DNase I (RNase free) kit (Fermentas, Life Sciences, UK). Total RNA (2 µg) was reverse-transcribed into cDNA in a reaction primed with random hexamer primers using the RevertAid H Minus First Strand cDNA Synthesis Kit (Fermentas, Life Sciences, UK) according to the manufacturer's recommendations. Polymerase chain reaction (PCR) primers for *TEX11, TEX12, TEX14*, and *TEX15* were designed using PerlPrimer v1.1.20 (Table 1).

**Table 1:** Oligonucleotide primer sequences

Gene	Forward primer	Reverse primer	Amplification	Product size with	NCBIReference sequence
			temperature (°C)	cDNA (bp)	gene
GAPDH	CTCATTTCCTGGTATGAC AACGA	CTTCCTCTTGTGCTCTTGCT	60	121	NG-007073.2
TEX11	GCCTGAATAGAGCCTTTGTGA	TAGATCAACTGCAACTGCCAT	62	250	NG-012574.1
TEX12	AGTCTCCAGTGCCAGATAGT	AGATTAATTTCCTTGCTCACATCA	62	135	NG-012574.1
TEX14	GGTTTATCCACCGCTCCCTC	CCTCTGTCCTCGCTTTCCAA	62	198	NG-012574.1
TEX15	AGGCAACATTCAAGCATCCA	AGTGAGCCAGGTAGTGATCTTT	62	141	NG-012574.1

After RNA extraction from testis tissue samples and cDNA synthesis, RT-PCR was performed with the related primers to confirm their expression in the samples of the three groups.

### Quantitative RT-PCR (RT-qPCR)

RT-qPCR was carried out to confirm and analyze the expression levels of target genes in the SCOS, MA, and control groups. The reactions were processed on a 7500 Real Time PCR machine (Applied Biosystems, Carlsbad, CA, USA) using the Power SYBR Green PCR master mix (Applied Biosystem, EU). The amplification solution contained 10 µl of Power SYBR Green PCR master mix, 50 ng of cDNA and 5 picomoles of each primer, yielding a final volume of 20 µl. Cycling conditions included an initial step of enzyme activation at 95°C for 10 minutes, followed by 40 cycles of denaturation at 95°C for 15 seconds, annealing at 62°C for 30 seconds, and extension at 72°C for 30 seconds. Transcript levels normalized to human glyceraldehyde 3-phosphate dehydrogenase (*GAPDH*) showed minimum variation among individual samples. *GAPDH* was used as an internal positive control. Each sample was run in duplicate and the mean value was calculated. No primer-dimer formation was observed during PCR amplification.

### Immunohistochemistry

The expression of *TEX14* was analyzed in the SO/H (n=3), SCOS (n=3), and MA (n=3) groups by immunohistochemistry. First, testis tissue sections were deparaffinized in xylene washes and then rehydrated in descending concentrations of ethanol washes. Endogenous peroxidase activity was blocked for 1 h at 37°C with goat serum (Sigma-Aldrich, St Louis, MO, USA), and triton X 100 was used to permeabilize cell membranes. Primary (*Tex14* Antibody, NBP1-85424 Novus, Biologicals USA, 1:100) and secondary antibodies (goat anti-rabbit HRP, ab97051, 1:100) were added and protein localization was visualized using a Dako Liquid DAB+ Substrate Chromogen System (Dako, Glostrup, Denmark). Finally, cells were imaged using an Olympus IX 71 microscope (Julaton & Pera, 2011). Negative controls were generated by the same method as the positive controls; however, the primary antibody was replaced with PBS solution.

### Statistical analysis

Data on clinical characteristics were shown as mean ± SEM. The normality of variables was analyzed with the Kolmogorov test. Differences in the mean values among the three groups were analyzed by one-way analysis of variance (ANOVA). The tests cited above were performed on SPSS statistical software package (SPSS Inc, Chicago, IL, USA) version 22.0. Real-time data were processed and analyzed using a two-tailed t-test. Differences with *p*-values <0.05 were considered significant.

## RESULTS

### Patient clinical characteristics

The characteristics of patients including age and LH, FSH, and testosterone levels are listed in Table 2. There was no significant difference in age, LH or testosterone serum levels between the three groups; however, the FSH serum levels between these groups were signiﬁcantly different (*p*=0.01).

**Table 2:** Clinical characteristics of patient groups. Values are expressed as mean±SEM

Patients groups	Age(years)	FSH(mIU/mL)	LH(mIU/mL)	Testosterone (ng/mL)
Maturation arrest (MA)	37.28±3.28(30-50)	5.62±1.51	4.32±1.26	5.28±0.84
Sertoli-cell-only syndrome (SCOS)	37.33±1.20(33-42)	18.57±3.90	3.73±1.82	3.27±0.59
Control	36.33±1.24(30-42)	8.37±1.73	3.70±0.87	5.61±3.57
*p*-value	0.924	0.010*	0.941	0.237

Values are expressed as mean±SEM.

* Significant difference based on ANOVA. Normal range of FSH: 1.5-12.4 mIU/mL Normal range of LH: 1.0-10.0 mIU/mL Normal range of Testosterone: 2.0-8.0ng/mL

### Gene expression analyses

**Figure 1:**
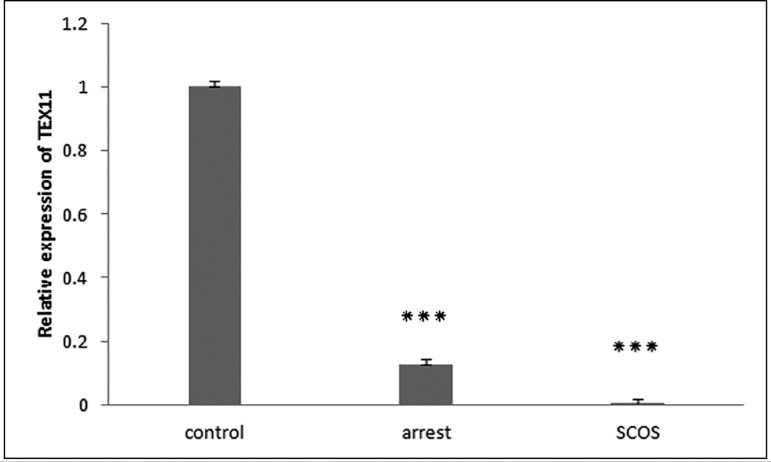
Comparison of the expression levels of *TEX11* between MA, SCOS, and control patients. *** *p*<0.05

**Figure 2:**
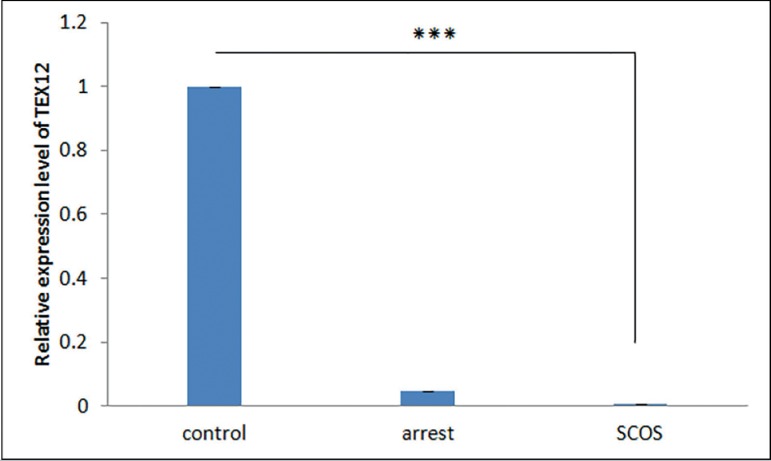
Comparison of the expression levels of *TEX12* between MA, SCOS, and control patients. *** *p*<0.05

**Figure 3:**
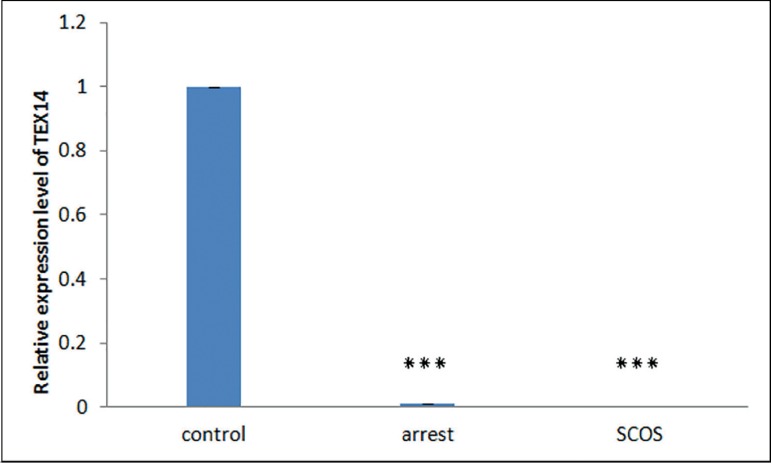
Comparison of the expression levels of *TEX14* between MA, SCOS, and control patients. *** *p*<0.05

**Figure 4:**
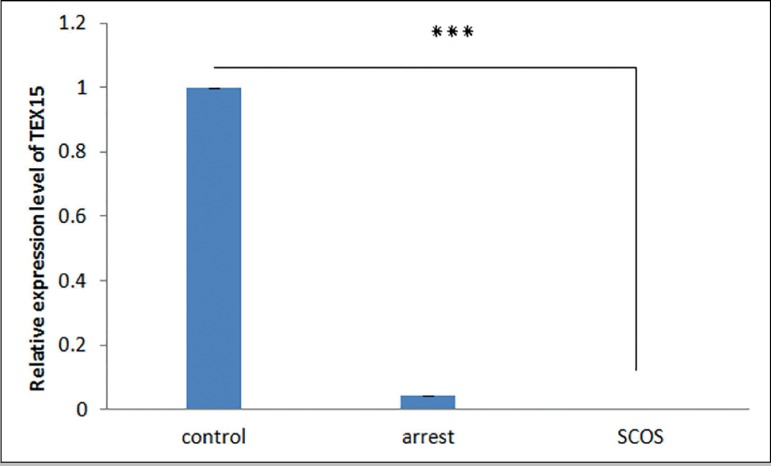
Comparison of the expression levels of *TEX15* between MA, SCOS, and control patients. *** *p*<0.05

The RT-qPCR results demonstrated that transcripts of all four *TEX* genes existed in all samples of the SCOS, MA, and control groups. Differential expression analysis showed that expression of *TEX* genes is significantly lower in SCOS testes samples when compared with controls (*p*<0.01) (Figures 1-4). The expression of *TEX11* and *TEX14* was also significantly lower in MA samples compared with controls (*p*≤0.038) (Figures 1,3); however, expression of *TEX12* and *TEX15* was not significantly different (*TEX12,15 p*>0.05) (Figures 2,4). Expression levels of *TEX12, TEX14* and *TEX15* in SCOS and MA samples were not significantly different (*TEX12*, 14,15 *p*>0.05) (Figures 2,3, and 4); however, *TEX11* expression was significantly lower in SCOS samples (*p*=0.003) (Figure 1).

### Immunohistochemical analysis of *TEX14* expression

**Figure 5:**
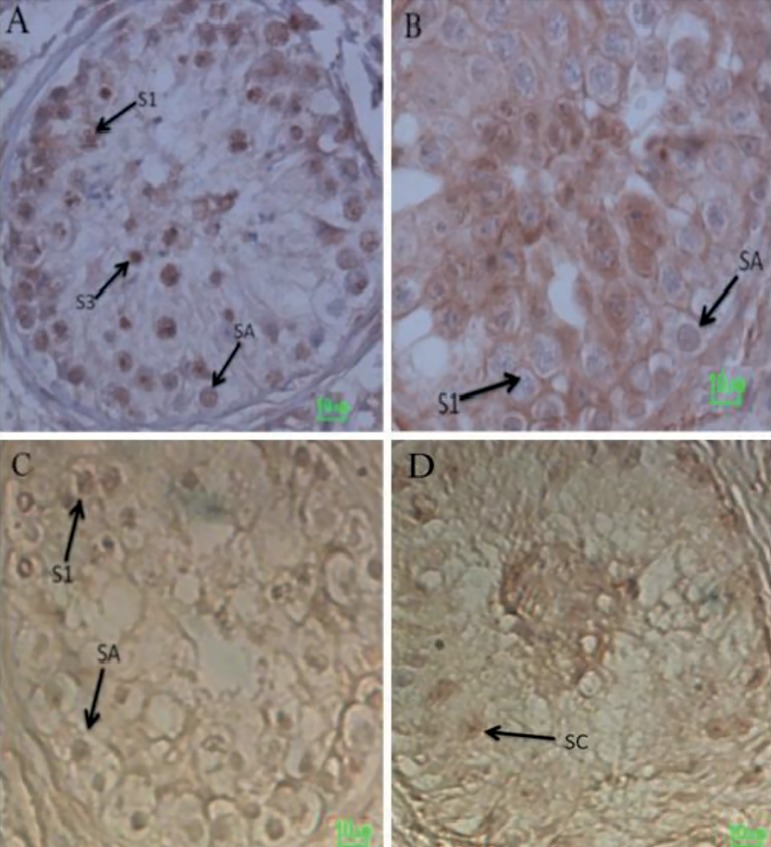
(A-D) Immunohistochemical analysis of adult testis sections with *TEX14* antibody. (A) *TEX14* is expressed in human testis cross-sections in obstructive azoospermia tissue samples. *TEX14* is expressed specifically by germ cells at varying stages of spermatogenesis. Type A spermatogonia (SA), primary spermatocytes (S1) and early round spermatids (S3) stain positively for *TEX14*. (B) *TEX14* is expressed in human testis cross-sections in MA tissue samples. (C) Negative control of testis cross-sections in MA tissue samples. (D) No positive signal is detected in adult human testis of SCOS tissue samples. The scale bar represents 10µm

Immunohistochemical analysis with *TEX14* antibody indicated absence of Leydig cell staining in several testis samples in all three groups. *TEX14* was however detected in a small number of Sertoli cells, averaging 1-2 positive cells per tubule, consistent with the low level of RNA detected by RT-qPCR. The intensity of staining in germ cells of control tissues was higher than in MA tissues (Figure 5). *TEX14* was expressed specifically by germ cells at varying stages of spermatogenesis. Type A spermatogonia (SA), primary spermatocytes (S1), and early round spermatids (S3) were positively stained for *TEX14*. No positive signal was detected in SCOS tissue samples.

## DISCUSSION

The same expression pattern of *TEX11, TEX12, TEX14* and *TEX15* genes in mice and humans indicates that these conserved genes may have an important role in the early stages of mammalian spermatogenesis *(**Zheng et al.,* 2010; Tang *et al*., 2011; Stouffs *et al.*, 2009; Greenbaum *et al*., 2006; Wu *et al*., 2003; Hamer *et al*., 2006; Yang *et al*., 2008a). Although the function of these genes in humans is not well-known, their roles in the progression of mouse spermatogenesis (Zheng *et al*., 2010; Greenbaum *et al*., 2006; Wu *et al.,* 2003; Hamer *et al*., 2006; Yang *et al*., 2008b) led us to hypothesize that they were likely to have the same roles in humans. To test this hypothesis, we compared the expression levels of these genes in testicular tissue samples of infertile men with two distinct histological patterns of spermatogenic failure (MA and SCOS) - our case group - to the levels observed in a control group including men with complete spermatogenesis (obstructive azoospermia).

Given that there was no germ cell in the testicular tissue samples of SCOS patients and all four *TEX* genes are germ cell-specific, down-regulation of these genes was expected. This observation is in line with the findings in mice, in which the lack of these genes disrupts the development of meiosis, resulting in male infertility (Zheng *et al.*, 2010; Greenbaum *et al.*, 2006; Wu *et al.*, 2003; Hamer *et al.*, 2006; Yang *et al*., 2008). The similar expression pattern and sequence conservation of *TEX11* seen in mice, humans, and pigs indicate that this gene is highly conserved and may have an important role in mammalian testicular function, including spermatogenesis (Zheng *et al*., 2010; Tang *et al*., 2011; Stouffs *et al*., 2009). *TEX11* expression in testis is correlated with the onset of spermatogenesis and is restricted to spermatocytes and round spermatids (Tang *et al.*, 2011). Stage-specific expression of *TEX11* in porcine meiotic germ cells is in agreement with findings in mice, where the lack of *Tex11* disturbs the progression of meiosis and thus results in infertility. The loss of function of *Tex11* in *Tex11-*null mice resulted in meiotic arrest and deletion of spermatocytes (Yang *et al.*, 2008b). In summary, the expression pattern of *Tex11* is highly preserved in rodents and higher mammals such as pigs and humans (Tang *et al.*, 2011).

Yang *et al.* (2015) demonstrated that the frequency of rare *TEX11* mutations is significantly higher in azoospermic men, suggesting that *TEX11* is essential for human spermatogenesis and mutations in this single X-linked gene is the cause of infertility in ~1% of azoospermic men. The authors also reported that the level of *TEX11* protein must be above a critical threshold for meiosis to progress, and that low-expressing *TEX11* alleles may thus result in human male infertility. Yatsenko *et al*. (2015) hypothesized that mutations in human *TEX11* disrupt the formation and function of the synaptonemal complex, resulting in disturbance of pachytene synapsis, meiotic arrest, and azoospermia. The authors reported that hemizygous *TEX11* mutations were a common cause of meiotic arrest and azoospermia in infertile men.

Our results showed that *TEX11* expression was significantly decreased in MA patients when compared to controls. As dysfunction of *TEX11* results in meiotic arrest and sterility in mice (Adelman & Petrini, 2008; Yang *et al*., 2008b; Stouffs *et al*., 2009), impairment of spermatogenesis and maturation arrest in this group may be linked to *TEX11* down-regulation. This down-regulation was significantly more pronounced in SCOS patients than in patients with MA. Our results confirmed the involvement of *TEX11* in human spermatogenesis. Furthermore, our findings indicated that expression of this gene is required for the completion of spermatogenesis.

*Tex12* expression is limited to meiotic cell division and its promoter, like many other germ cell-specific genes, is quenched in somatic cells by transcription factor E2F6 (Pohlers *et al*., 2005). Previous studies showed that *TEX12* localizes to the central element of the synaptonemal complex. Their results also revealed that without *TEX12*, the synaptonemal complex lacks a correct central element structure, synapsis cannot complete, and meiotic recombination and crossing-over do not occur (Hamer *et al*., 2006). Since loss of *TEX12* results in the inaccurate assembly of the synaptonemal complex and non-chromosomal synapsis (Hamer *et al*., 2006), reduced expression of this gene in the MA group in relation to the control group was expected; however, our results did not reveal a significant difference in expression between these two groups.

Previous studies of *Tex15* in mice showed this gene is necessary for chromosomal synapsis, DSB repair, and meiotic recombination during meiosis; therefore, *TEX15* is thought of as a possible factor in spermatogenic failure risk (Yang *et al.*, 2008a). Okutman *et al.* (2015) showed that a nonsense mutation in *TEX15* is the cause of spermatogenic defect in familial cases of teratozoospermia and infertility. Immunohistochemistry confirmed these findings showing high levels of expression in germ cells and low levels of expression in Sertoli cells. The tests also revealed that *Tex15* knock-out produced spermatogenesis meiotic arrest and severe reduction in testicular size. Therefore, we expected to observe down-regulation of *TEX15* in the MA group when compared to controls. However, our results did not show any significant difference. This may possibly be due to high levels of expression in spermatogonia and early spermatocytes in the testicular tissue of MA patients. Previous studies have shown spermatogenic disruption in *Tex14* knockout mice before the first meiotic division due to loss of intercellular bridges (Bolcun-Filas *et al*., 2009; Greenbaum *et al*., 2006; 2009). Moreover, a study on *Tex14* mutant pigs showed that *Tex14* is also essential for the completion of spermatogenesis in pigs (Sironen *et al*., 2011). Although *TEX14* localizes to intercellular bridges of both female and male mice germ cells, disruption of this gene leads to sterility only in males (Brill *et al.*, 2000; Greenbaum *et al*., 2006). The comparison of *TEX14* expression between control and MA groups showed that *TEX14* expression had a significant decrease in MA samples. As dysfunction of *TEX14* results in meiotic arrest and sterility in mice (Greenbaum *et al.*, 2006; 2009; Wu *et al*., 2003), impairment of spermatogenesis and maturation arrest may be linked to the down-regulation of *TEX14*. These results were confirmed by immunohistochemistry, with *TEX14* expressed in type A spermatogonia, primary spermatocytes, and early round spermatids in control samples. Conversely, this protein was absent or hardly visible in type A spermatogonia, primary spermatocytes, and early round spermatids of MA samples. These results support the possibility that *TEX14* under-expression in MA patients is etiologic, suggesting that the low expression of this protein in these patients is unlikely to be caused by the lack of germ cells.

## CONCLUSION

This study demonstrated that *TEX11, TEX12, TEX14* and *TEX15* are essential for the completion of spermatogenesis. Down-regulation of these genes, especially *TEX14* and *TEX11*, may lead to impaired spermatogenesis in infertile men. Studies on other independent sample sets are required to confirm this association.
